# Letter to the editor: High-frequency ultrasound is an effective adjunct for lymphatic surgery

**DOI:** 10.1016/j.jpra.2026.02.008

**Published:** 2026-02-11

**Authors:** David Kwan Ryung Lee, Dave Osinachukwu Duru, Kai Yuen Wong

**Affiliations:** a School of Clinical Medicine, University of Cambridge, Hills Rd, Cambridge, UK; b Department of Plastic and Reconstructive Surgery, Cambridge University Hospitals NHS Foundation Trust, Cambridge, UK

**Keywords:** High-frequency ultrasound, Lymphatic surgery, Lymphaticovenous anastomosis, Lymphoedema, Supermicrosurgery

Dear Editor,

There is increasing evidence to support the use of lymphaticovenous anastomosis (LVA) surgery, for the prevention and treatment of lymphoedema. Indocyanine green (ICG) lymphography is the current gold standard imaging modality used for pre-operative mapping of lymphatic channels.

High-frequency ultrasound (HFUS) is a promising adjunct. Ultrasound typically operates within a frequency range of 1–15 MHz. Although no universal consensus exists, HFUS is defined as the use of probes at the upper end of this range – commonly 15 MHz and above. 15 MHz probes can provide exceptionally high spatial resolutions of up to 0.23 mm in real-time and are therefore effective for pre-operatively assessing the location and quality of lymphatic vessels, typically 0.3–0.8 mm in diameter. The ability of HFUS to successfully identify these vessels has been well documented but there is limited literature on its outcomes. Tables 1 and 2 present evidence indicating that HFUS assisted LVA surgery improves patient results – lymphoedema indices and cellulitis frequencies respectively.^1^, ^2^, ^3^, ^4^ All studies used lymphoscintigraphy to initially diagnose or grade the severity of lymphoedema, before using ICG lymphography to produce a general map of the lymphatic vessels and HFUS to assess specific vessels for surgery. Hashem and Yamamoto^2^ was an exception and used HFUS exclusively for pre-operative LVA planning. Malagón and Yamamoto^3^ and Hashem and Yamamoto^2^ were the only studies to report subjective outcomes, and both found improvements in the Lymphoedema Quality of Life Score (*p* < 0.001) and limb tension/heaviness/mobility in the majority of patients respectively.

**Table 1:** Changes in lymphoedema indices after HFUS assisted lymphatic surgery.

Author	Year	Limb site (*n*)	Lymphatic vessel quality	Mean pre-operative lymphoedema index	Mean post-operative lymphoedema index	Mean change in lymphoedema index (%)	*p*-value
Bianchi et al.^1^	2020	Upper limb (9)	High	138.63 ± 32.48	126.65 ± 32.43	–8.6	**0.026**
Bianchi et al.^1^	2020	Lower limb (7)	High	348.09 ± 59	308.20 ± 29.85	–11.5	**0.001**
Bianchi et al.^1^	2020	Upper limb (5)	Low	122.28 ± 33.31	114.84 ± 27.43	–6.1	0.135
Bianchi et al.^1^	2020	Lower limb (5)	Low	298.59 ± 35.8	285.14 ± 36.31	–4.5	**0.032**
Hashem and Yamamoto^2^	2025	Upper limb (23)	Not specified	136.4 ± 9.4	123.5 ± 7.3	–9.5	**0.017**
Malagón and Yamamoto^3^	2025	Lower limb (97)	Not specified	281.2 ± 31.5	267.6 ± 28.7	–4.8	**0.002**

Key: Lymphoedema index – Quantitative measurement of limb swelling calculated from serial limb circumference measurements.

**Table 2:** Changes in cellulitis frequencies after HFUS assisted lymphatic surgery.

Author	Year	Pre-operative cellulitis frequency (/yr)	Post-operative cellulitis frequency (/yr)	Change in cellulitis frequency (%)	Significance
Rodriguez and Yamamoto^4^	2022	2.1	0.2	–90.5	***p*<0.05**
Hashem and Yamamoto^2^	2025	2.15	0.09	–95.8	***p*<0.001**

Bianchi et al.^1^ found that HFUS could accurately evaluate the quality of lymphatic vessels, as confirmed by histological analysis, in addition to improving patient outcomes. They found that performing LVA surgery on less sclerotic lymphatic vessels was associated with greater improvements in lymphoedema indices, when compared to cases where LVAs performed on more sclerotic vessels. Note only the decrease in the lower limb lymphoedema index was statistically significant in the latter instance (Table 1).

Although emerging evidence supports the use of HFUS in lymphatic surgery, studies comparing outcomes of planning LVA surgery with and without ICG lymphography, with or without HFUS assistance are required. It is interesting to note that Hara and Mihara^5^ found that even moderately high frequency probes of 10–12 MHz showed a high success rate in identifying lymphatic vessels, particularly with regards to the lower limb (89.5%). Either way, the use of ultrasound will be operator dependent with a learning curve. More widespread adoption of this modality for lymphatic surgery planning will also require incorporation into plastic surgery curricula.

## Declaration of competing interest

None declared.
